# Screening and Identification of Key Biomarkers in Acquired Lapatinib-Resistant Breast Cancer

**DOI:** 10.3389/fphar.2020.577150

**Published:** 2020-09-04

**Authors:** Shengnan Bao, Yi Chen, Fan Yang, Chunxiao Sun, Mengzhu Yang, Wei Li, Xiang Huang, Jun Li, Hao Wu, Yongmei Yin

**Affiliations:** ^1^Department of Oncology, The First Aﬃliated Hospital of Nanjing Medical University, Nanjing, China; ^2^The First Clinical College of Nanjing Medical University, Nanjing, China; ^3^Jiangsu Key Lab of Cancer Biomarkers, Prevention and Treatment, Collaborative Innovation Center for Personalized Cancer Medicine, Nanjing Medical University, Nanjing, China

**Keywords:** HER2-positive breast cancer, acquired lapatinib resistance, biological markers, hub genes, prognostic analysis

## Abstract

Lapatinib, targeting the human epidermal growth factor receptor family members HER1 and HER2, has been approved by the US Food and Drug Administration for use in metastatic HER2-positive breast cancer. However, resistance to lapatinib remains a common challenge to HER2-positive metastatic breast cancer. Until now, the molecular mechanisms of acquired resistance to lapatinib (ALR) have remained unclear. With no definite biomarkers currently known, we aimed to screen for key biomarkers in ALR. In this research, we identified 55 differentially expressed genes (DEGs, 20 upregulated, 35 downregulated) through bioinformatic analysis using microarray datasets GSE16179, GSE38376, and GSE51889 from the Gene Expression Omnibus (GEO) database. The related gene function was explored using the Gene Ontology (GO) function and Kyoto Encyclopedia of Genes and Genomes (KEGG) pathway enrichment analysis. The protein-protein interaction (PPI) network was constructed with the Search Tool for the Retrieval of Interacting Genes (STRING) and Cytoscape. The functional enrichment of the DEGs was analyzed, including negative regulation of the B cell apoptotic process, DNA replication, solute:proton symporter activity, synthesis, and degradation of ketone bodies, and metal sequestration by antimicrobial proteins. Analysis of seven hub genes revealed their concentration mainly in DNA replication and cell cycle. Survival analysis revealed that *MCM10* and *SPC24* may be related with poor prognosis in patients with ALR. Meanwhile, the prediction model of lapatinib sensitivity was constructed, and emerging role of the model was further analyzed using several webtools. In conclusion, hub genes are involved in the complex mechanisms underlying ALR in breast cancer and provide favorable support for treatment of ALR in future.

## Introduction

Based on molecular markers, breast cancer is divided into four subgroups: luminal A, luminal B, basal-like, and human epidermal growth factor receptor 2 (HER2)-enriched (Perou et al., 2000). Receptor tyrosine-protein kinase HER2, also known as erbB-2, is included in the epidermal growth factor receptor (EGFR) family of receptor tyrosine kinases (Oh and Bang, 2020). HER2 is overexpressed in 20%–25% of breast cancer patients. HER2 over-expression is known as an aggressive tumor phenotype and is associated with worse survival (Parakh et al., 2017). Lapatinib, a reversible tyrosine kinase inhibitor with specificity for both EGFR and HER2, is approved for treating HER2-positive metastatic breast cancer after disease progression with trastuzumab therapy (Gradishar, 2013; Moasser and Krop, 2015). Compared with capecitabine monotherapy, lapatinib in combination with capecitabine improved objective response rate and progression-free survival (Geyer et al., 2006). Despite the effectiveness of lapatinib in HER2-positive breast cancer, acquired resistance remains a major clinical obstacle. D’Amato et al. have pointed out multiple mechanisms of ALR in breast cancers, including activation of compensatory pathways, mutation of the HER2 kinase domain, and gene amplification (D’Amato et al., 2015). Critically, there are currently no definite biomarkers to predict patients’ responses to lapatinib.

With the development of gene sequencing and bioinformatics, increasing number of genetic studies have revealed the mechanism of tumorigenesis and drug resistance. By introducing microarray data and bioinformatic analysis that have been widely applied to investigate whole expression of genes in cancer, researchers have deepened their understanding of the differentially expressed genes (DEGs) and functional enrichment analysis among the complex diseases (Zhu et al., 2017). Although there are some bioinformatic studies corresponding to resistance to anti-HER2 therapies, scarce data and different laboratory conditions make it difficult to acquire reliable results. To overcome the limitation of insufficient data, we identified DEGs through bioinformatic analysis with three Gene Expression Omnibus (GEO) microarray datasets. Additionally, the related gene function was explored with Gene Ontology (GO) function and Kyoto Encyclopedia of Genes and Genomes (KEGG) pathway enrichment analysis, and the protein-protein interaction (PPI) network was determined to express functions to establish a solid theoretical framework for potential molecular mechanisms. In the present study, we aimed to screen for biomarkers in ALR and found that there were 55 DEGs and 7 hub genes, which may be potential biological markers and provide a theoretical support for further treatment of ALR. The specific prediction model was established to evaluate the relationship between clinicopathologic characteristics of breast cancer patients and sensitivity of lapatinib.

## Materials and Methods

### Selection of Microarray Datasets

**Figure 1:**
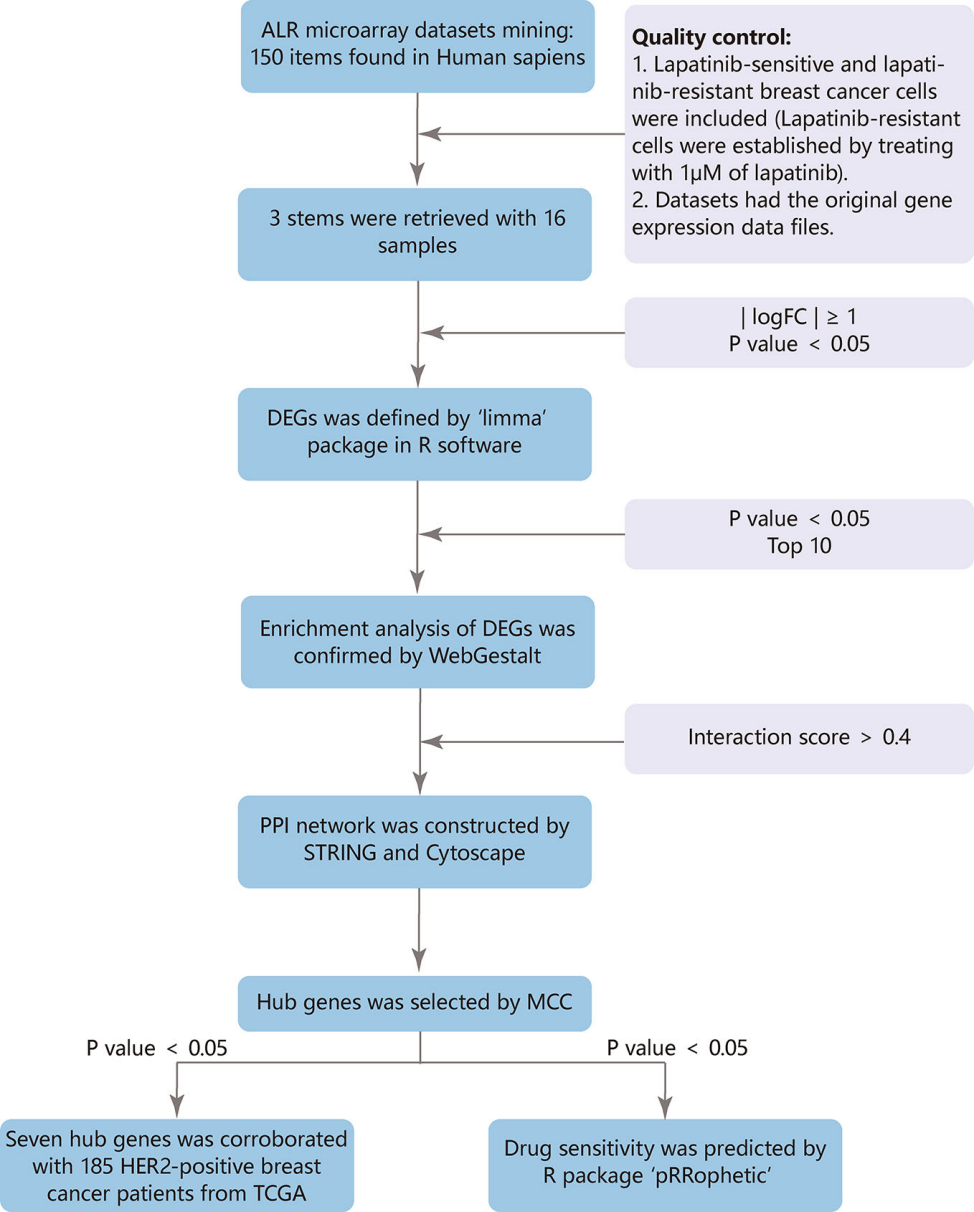
Flow chart.

The GEO (http://www.ncbi.nlm.nih.gov/geo) provides a public platform to obtain different datasets from high-throughput gene expression and genomic hybridization experiments, including platforms, samples, and series (Edgar et al., 2002). We chose the key words “lapatinib resistant” and the organism ‘Homo sapiens’ (**Figure 1**). Three gene expression datasets [GSE16179 (Liu et al., 2009), GSE38376 (Komurov et al., 2012), GSE51889 (Chen et al., 2013)] were selected from GEO. GSE16179 contained 3 lapatinib-sensitive cell samples and 3 lapatinib-resistant cell samples. GSE38376 contained 3 lapatinib-sensitive cell samples and 3 lapatinib-resistant cell samples. GSE51889 contained 2 lapatinib-sensitive cell samples and 2 lapatinib-resistant cell samples. To establish lapatinib-resistant cells, BT474 and SKBR3 were treated with 1 μM of lapatinib (**Table 1**). The series matrix file and platform were downloaded to convert the probes into the corresponding gene symbol, using Practical Extraction and Report Language (Perl) (https://www.perl.org/) scripts.

**Table 1:** Characteristics of individual studies selected from GEO.

Dataset	Platform	Samples (Cancer cell)		Drug
		Lapatinib-sensitive	Lapatinib-resistant	
GSE16179	GPL570	3 (BT474)	3 (BT474-J4)	Lapatinib
GSE38376	GPL6947	3 (SKBR3)	3 (SKBR3-R)	Lapatinib
GSE51889	GPL6480	2 (SKBR3、BT474)	2 (SKBR3,lapatinib-resistant、BT474,lapatinib-resistant)	Lapatinib

GEO, Gene Expression Omnibus. BT474、SKBR3: an HER2-positive and lapatinib-sensitive cell line. BT474-J4、SKBR3-R: an acquired lapatinib resistant cell line.

### Definition of DEGs

Three datasets were merged using Perl scripts to obtain more genes fully. To reduce deviation in data processing, we normalized batch effect using “sva” package in R software and screened the DEGs between lapatinib-sensitive cell samples and lapatinib-resistant cell samples using the “limma” package in R software (http://www.r-project.org/) (Davis and Meltzer, 2007). The difference was considered significant when |logFC| (fold change) was ≥ 1 and P value was < 0.05.

### Enrichment Analysis of DEGs

The web-based Gene SeTAnaLysis Toolkit (WebGestalt) (http://www.webgestalt.org/) is one of the most widely used online databases that helps researchers extract biological information from genes of interest (Liao et al., 2019). The goal of GO is to provide a vocabulary that can be applied to the shared genes and proteins, annotating genes, and analyzing biological process (Ashburner et al., 2000). KEGG is a database that sheds light on higher-order functional behaviors from molecular information generated by genome sequencing and other high-throughput experimental techniques (Kanehisa, 2002). Reactome functions as an extended version of a classic metabolic map (Jassal et al., 2020). To confirm characteristic biological functions of DEGs, analyses were performed using WebGestalt. The difference was considered significant when P value was < 0.05 and top 10 would be selected.

### Construction of PPI Network

The PPI network was utilized to reveal many functional relationships and interactions among predicted target proteins using the Search Tool for the Retrieval of Interacting Genes (STRING) (version 11.0) database (http://string-db.org) (Szklarczyk et al., 2015). An interaction score > 0.4 was considered statistically significant, and disconnected nodes in the network were hidden. Cytoscape (version 3.7.2) is a bioinformatics software platform for visualizing modules of the PPI network (Smoot et al., 2011). The cytoHubba of Cytoscape is an application for exploring important hubs and clustering an interactome network with topological algorithms (Chin et al., 2014). For each module, the GO, KEGG, and reactome analysis were performed using WebGestalt.

### Selection of Hub Genes

The hub genes were selected with the Maximal Clique Centrality algorithm (MCC). A network of their co-expressed genes was appraised by GeneMANIA (http://genemania.org/), which can provide gene function and extend the list with similar genes (Warde-Farley et al., 2010). Efficient hierarchical cluster analysis of hub genes was performed using UCSC Cancer Genomics Browser (http://xena.ucsc.edu/) (Kent et al., 2002). To perform survival analysis in a larger number of patients, gene expression data and clinicopathologic data of breast cancer patients were downloaded from The Cancer Genome Atlas (TCGA) at the UCSC Cancer Genomics Browser (http://xena.ucsc.edu/). The survival analysis of hub genes was constructed by the R packages “survival” and “survminer”. The difference was considered significant when P value was < 0.05.

### Prediction of Drug Sensitivity

Based on the data from TCGA of patients diagnosed with breast cancer, and drug sensitivity data from the Cancer Genome Project (CGP), the prediction model was performed using R package “pRRophetic” (Garnett et al., 2012; Geeleher et al., 2014a). The half-maximal inhibitory concentration (IC50) of lapatinib in each treated patient was obtained by ridge regression, and the prediction accuracy was measured through 10-fold cross-validation. The default parameters were chosen, including “combat” for removing the batch effect, “allSolidTumors” for tissue type, and mean value for identifying the duplicate gene expression (Geeleher et al., 2014b). The median IC50 was selected for risk stratification to develop a specific model: lower IC50 was more sensitive to lapatinib. GraphPad Prism 7.0 was used to analyze data, and categorical data was compared by chi-square test. The difference was considered significant when P value was < 0.05.

## Results

### Definition of DEGs in ALR

**Figure 2:**
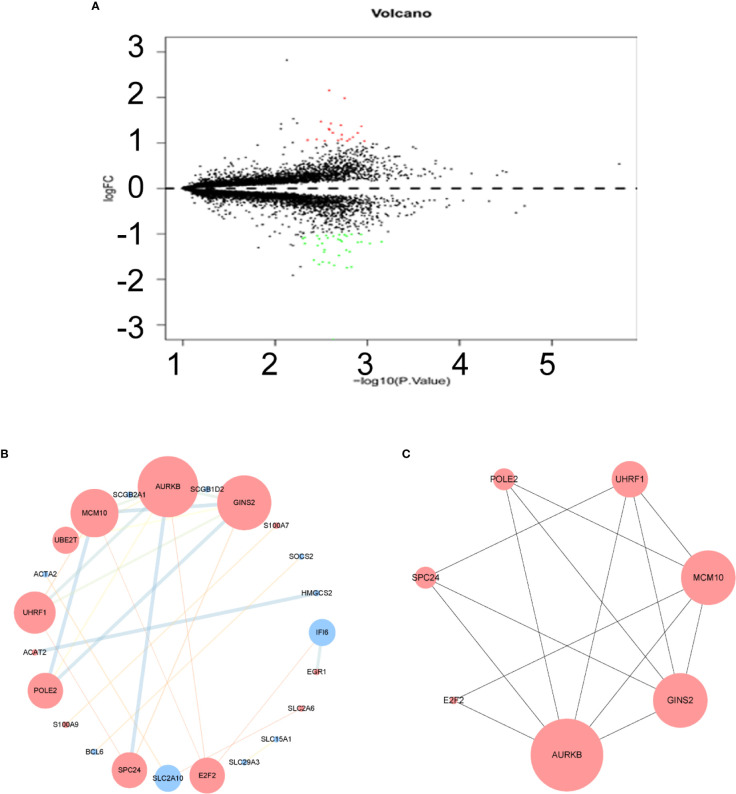
Volcano plot, protein-protein interaction (PPI) network and the most significant module of differentially expressed genes (DEGs). **(A)** DEGs were selected with |logFC|(fold change) ≥ 1 and P value < 0.05 among GSE16179, GSE38376, GSE51889. Upregulated genes are marked in red; downregulated genes are marked in green. **(B)** The PPI network of DEGs was constructed using Cytoscape. Map Node Size to degree, low values to small sizes; upregulated genes are marked in red and downregulated genes are marked in blue. Map Edge Size (Color) to combined score, low values to small sizes (bright colors). **(C)** The most significant module was obtained from PPI network with 7 nodes and 14 edges. Top 7 nodes are ranked by Maximal Clique Centrality algorithm (MCC).

After normalization of the microarray data, 14,653 genes were merged among three datasets. Fifty-five DEGs are shown in the volcano plot (**Figure 2A**), consisting of 20 upregulated genes and 35 downregulated genes between lapatinib-sensitive cells and lapatinib-resistant cells.

### Enrichment Analysis of DEGs

**Figure 3:**
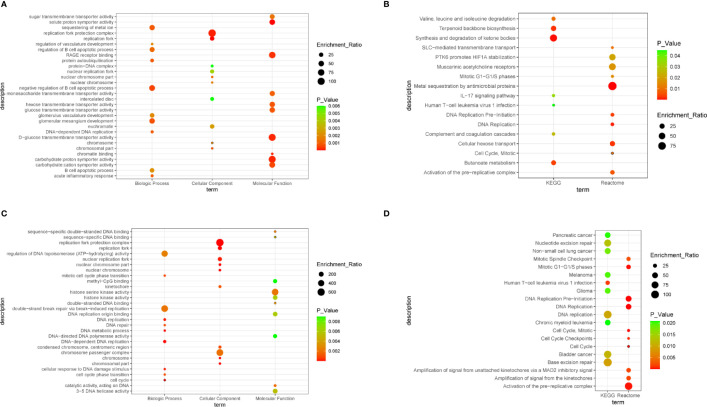
Bubble plot. **(A)** The significantly enriched gene ontology (GO) terms of differentially expressed genes (DEGs), with P value < 0.05. **(B)** Gene networks identified through Kyoto Encyclopedia of Genes and Genomes (KEGG) and Reactome analysis of DEGs, with P value < 0.05. **(C)** The significantly enriched GO terms of hub genes, with P value < 0.05. **(D)** Gene networks identified through KEGG and Reactome analysis of hub genes, with P value < 0.05.

To obtain a comprehensive understanding of the biological effect of 55 DEGs, WebGestalt was applied to describe functional enrichment (**Supplementary Table 1**). GO analysis indicated that negative regulation of B cell apoptotic process, glomerular mesangium development, sequestering of metal ion, DNA-dependent DNA replication, and protein autoubiquitination were the top 5 relevant biological process (**Figure 3A**). For the cellular component, replication terms were mainly enriched (**Figure 3A**). Molecular functions were concentrated mainly on solute:proton symporter activity, chromatin binding, and carbohydrate:proton symporter activity (**Figure 3A**). KEGG pathway analysis revealed that synthesis and degradation of ketone bodies, terpenoid backbone biosynthesis, and butanoate metabolism were three of the most enriched pathways (**Figure 3B**). Reactome pathway analysis demonstrated that DEGs were mainly enriched in metal sequestration by antimicrobial proteins, DNA replication, and cellular hexose transport (**Figure 3B**).

### Construction of PPI Network

To understand the biological activity at the protein level, an integrated PPI network of these DEGs was performed (**Figure 2B**), and the most important module was constructed using Cytoscape (**Figure 2C**, **Supplementary Table 2**). Genes in the module were concentrated mainly on cell cycle and DNA replication (**Figures 3C, D**, **Supplementary Table 3**).

### Selection of Hub Genes

**Figure 4:**
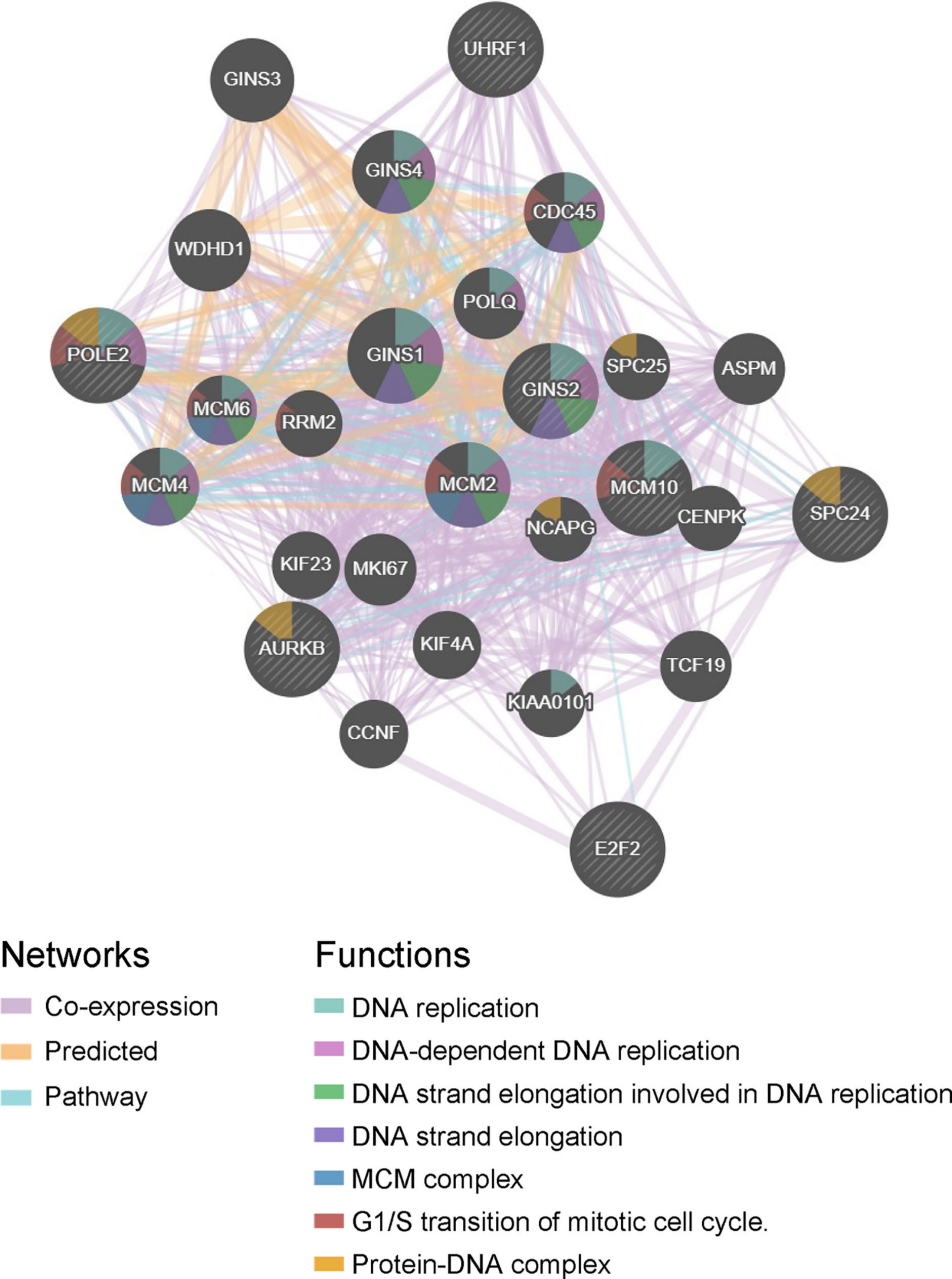
Interaction network. A network of the hub genes and their co-expressed genes was analyzed using GeneMANIA.

**Figure 5:**
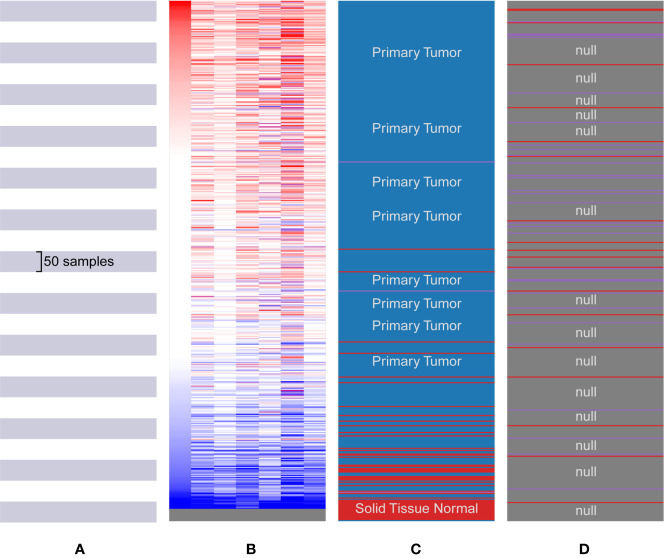
Biological process analysis of the hub genes. **(A)** The Cancer Genome Atlas (TCGA) samples. **(B)** Upregulation of genes is marked in red; downregulation of genes is marked in blue. **(C)** Hierarchical clustering of hub genes was constructed using UCSC. The samples under the red bar are normal solid tissues and the samples under the blue bar are primary tissues. **(D)** Positive metastatic breast cancinoma estrogen receptor are marked in purple; negative in red.

**Figure 6:**
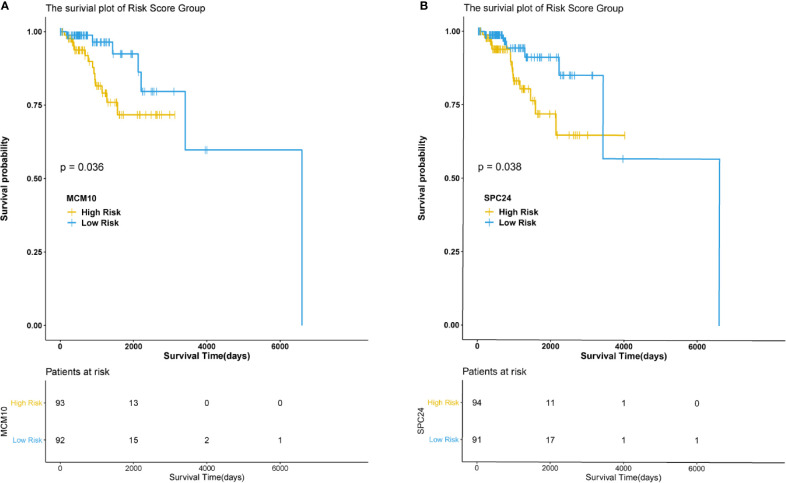
Survival analysis of the hub genes. P < 0.05 was considered statistically signifificant. **(A)** Overall survival by low and high MCM10 expression. **(B)** Overall survival by low and high SPC24 expression.

The hub genes were calculated by Matthews Correlation Coefficient (MCC), and the top seven genes were selected. A network of their co-expressed genes was interpreted by GeneMANIA (**Figure 4**). The hub genes could distinguish breast cancer samples from normal tissue samples through hierarchical clustering (**Figures 5A–C**). When the hub genes were expressed highly, an increasing number of samples presented estrogen receptor status (**Figure 5D**). The survival analysis of hub genes from TCGA was verified in 185 patients diagnosed with HER2-positive breast cancer. Patients with high *MCM10* and *SPC24* expression showed worse overall survival (**Figure 6**).

### Prediction of Drug Sensitivity

**Figure 7:**
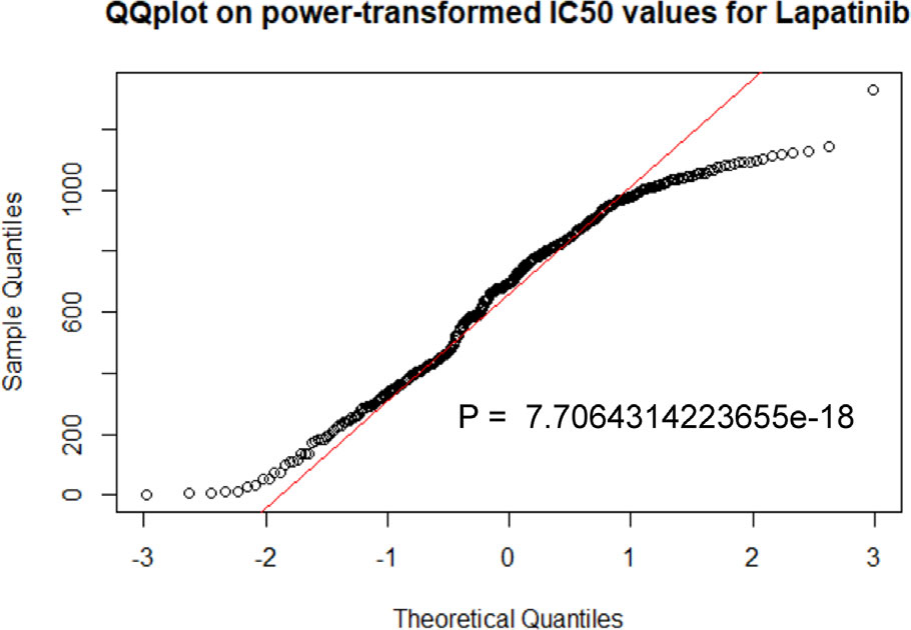
The prediction model was performed by R package “pRRophetic”. P < 0.05 was considered statistically significant.

The specific prediction model could evaluate the relationship between breast cancer patients and sensitivity of lapatinib (**Figure 7**, **Table 2**). A cohort involving 664 breast cancer patients with clinicopathologic data from TCGA was analyzed. The distributions of patients’ age and gender were not significantly different between the low-IC50 and high-IC50 groups. The high-IC50 group tended to include more non-white patients (chi-square test, P = 0.03) and more patients with stage II cancer (chi-square test, P = 0.0003). More patients with infiltrating lobular carcinoma and mixed histology were included in low-IC50 group (chi-square test, P = 0.0004). Interestingly, there were more patients with both HER2-positive and hormone receptor-positive and luminal subtype in low-IC50 group (chi-square test, P < 0.0001) indicating that the status of hormone receptor could influence lapatinib sensitivity.

**Table 2:** Clinicopathologic characteristics of patients in different risk groups in TCGA BRCA cohort.

Characteristics	Whole cohort(n = 664)	Low-IC50(n = 332)	High-IC50(n = 332)	P value
**Age**				0.55
< 50 years	192 (28.9%)	92 (27.7%)	100 (30.1%)	
≥50 years	472 (71.1%)	240 (72.3%)	232 (69.9%)	
**Gender**				0.70
Female	7 (1.1%)	4 (1.2%)	3 (0.9%)	
Male	657 (98.9%)	328 (98.8%)	329 (99.1%)	
**Race**				0.03
Asian	38 (5.7%)	16 (4.8%)	22 (6.6%)	
Black	137 (20.6%)	56 (8.4%)	81 (24.4%)	
White	438 (66.0%)	232 (69.9%)	206 (62.1%)	
**Pathologic tumor stage**				0.0003
Stage I	111 (16.7%)	63 (19.0%)	48 (14.5%)	
Stage II	387 (58.3%)	168 (50.6%)	219 (66.0%)	
Stage III	147 (22.1%)	92 (27.7%)	55 (16.6%)	
Stage IV	9 (1.4%)	6 (1.8%)	3 (0.9%)	
**Histological type**				0.0004
Infiltrating Ductal Carcinoma	477 (71.8%)	223 (67.2%)	254 (76.5%)	
Infiltrating Lobular Carcinoma	126 (19.0%)	84 (25.3%)	42 (12.7%)	
Medullary Carcinoma	6 (0.9%)	0 (0)	6 (1.8%)	
Mucinous Carcinoma	10 (1.5%)	3 (0.9%)	7 (2.1%)	
Mixed Histology	15 (2.3%)	9 (2.7%)	6 (1.8%)	
Metaplastic Carcinoma	8 (1.2%)	3 (0.9%)	5 (1.5%)	
Other	21 (3.2%)	10 (3.0%)	11 (3.3%)	
**Molecular subtype**				<0.0001
HER2+, HR-	13 (2.0%)	5 (1.5%)	8 (2.4%)	
HER2+, HR+	64 (9.6%)	45 (13.6%)	19 (5.7%)	
TNBC	165 (24.9%)	27 (8.1%)	138 (41.6%)	
Luminal	395 (59.5%)	246 (74.1%)	149 (44.9%)	

TCGA, The Cancer Genome Atlas; BRCA, breast cancer; IC50, half-maximal inhibitory concentration; HR, hormone receptor; TNBC, triple-negative breast cancer.

Patients with information unavailable on race (51 patients, 7.7%), pathologic tumor stage (10 patients, 1.5%), histological type (1 patient, 0.1%), molecular subtype (27 patients, 4.0%) were excluded from the comparison.

## Discussion

Although lapatinib has been approved for treating HER2-positive metastatic breast cancer after trastuzumab failure, acquired resistance to lapatinib (ALR) remains a major clinical challenge. However, the molecular mechanisms of ALR remain unclear. Thus, in-depth study of the mechanism of ALR and discovery of biomarkers with high sensitivity and specificity are of great value to improve the prognosis of patients with HER2-positive breast cancer. Microarray data and bioinformatic analysis have enabled us to explore the whole expression of genes in ALR and improved our understanding of DEGs and functional pathways in complex diseases.

In the current study, DEGs were analyzed using three mRNA microarray datasets between lapatinib-sensitive cell samples and lapatinib-resistant cell samples. Fifty-five DEGs were analyzed, consisting of 20 upregulated genes and 35 downregulated genes. Functional enrichment analysis of GO and KEGG revealed that the majority of DEGs were associated with the following cellular processes: cell cycle, development, apoptosis, and signal transduction. In particular, several terms, including GO terms “negative regulation of B cell apoptotic process”, “replication fork”, “solute:proton symporter activity”, KEGG terms “synthesis and degradation of ketone bodies”, “terpenoid backbone biosynthesis”, and reactome terms “metal sequestration by antimicrobial proteins”, “DNA replication”, were closely related with classic mechanisms of ALR in breast cancers. The hub genes were calculated by MCC, and the top seven genes including *AURKB*, *GINS2*, *MCM10*, *UHRF1*, *POLE2*, *SPC24*, and *E2F2* were presumed to be associated with drug resistance. Seven hub genes were later corroborated with TCGA data from 185 HER2-positive breast cancer patients. Survival analysis showed that *MCM10* and *SPC24* may be related with poor prognosis in patients with acquired lapatinib resistance. Furthermore, the specific prediction model revealed that the distributions of patients’ races, pathologic tumor stages, histological types, and molecular subtypes were significantly different between low-IC50 and high-IC50 groups.

MCM10, a highly conservative mini-chromosome maintenance protein, is involved in the initiation of eukaryotic genome replication. In a recent study, MCM10 induced migration and invasion of breast cancer *via* the Wnt/β-catenin pathway (Yang and Wang, 2019). SPC24, a component in the kinetochore microtubule interface, is associated with tumorigenic transformation (Zhu et al., 2015). Additionally, SPC24—which monitors the PI3K/AKT kinase pathway—is overexpressed in breast cancer, implying its importance in clinical treatment (Zhou et al., 2018). Although E2F2 was not found to be of significance in survival analysis, it could be a gene of interest. E2F2, a member of the E2F family, is typically repressed by the retinoblastoma protein pRB. E2F transcription factors exist in the CDK4/6-RB1 pathway, and this pathway is often dysregulated in hormone receptor-positive breast cancer (Spring et al., 2020). Many researches have reported that E2F1-3 transcripts are highly expressed in HER2-positive tumors (Andrechek, 2015; Wu et al., 2015). Nikolai et al. found that the potential for combined targeting of HER2 and CDK signaling pathways may be a prospective strategy (Nikolai et al., 2016). Therefore, CDK4/6 inhibitors may overcome resistance to lapatinib.

Overall, seven hub genes were analyzed, and results showed that these genes were concentrated mainly on DNA replication and cell cycle. Most interestingly, after analyzing the hub genes, we found that several signaling pathways may be related with ALR. Thus, many targeted drugs could be expected to reverse ALR.

## Conclusion

In conclusion, the overexpression of *AURKB*, *GINS2*, *MCM10*, *UHRF1*, *POLE2*, *SPC24*, and *E2F2* in HER2-positive breast cancer patients with ALR showed that these hub genes could be potential prognostic biomarkers in such patients. Survival analysis revealed that high *MCM10* and *SPC24* expression were negative prognostic factors in patients with acquired lapatinib resistance. Future preclinical and translational studies should be directed at defining mechanisms involved in ALR and a combination of targeted agents.

## Data Availability Statement

The data analyzed in this paper were obtained from the microarray datasets GSE16179, GSE38376, and GSE51889 from the Gene Expression Omnibus (GEO) database (http://www.ncbi.nlm.nih.gov/geo). Gene expression data and clinicopathologic data of breast cancer patients were downloaded from The Cancer Genome Atlas (TCGA) at the UCSC Cancer Genomics Browser (http://xena.ucsc.edu/).

## Author Contributions

YY designed the study. SB and YC conducted all statistical analyses and drafted the manuscript. FY, CS, MY, WL, XH, JL, and HW revised the manuscript. All authors contributed to the article and approved the submitted version.

## Funding

This study was financially supported by the 333 Project of Jiangsu Province (BRA2017534 and BRA2015470), the High-level innovation team of Nanjing Medical University (JX102GSP201727), the Key medical talents (ZDRCA2016023), the National Key Research and Development Program of China (ZDZX2017ZL-01), the Project of China key research and development program precision medicine research (2016YFC0905901), the collaborative innovation center for tumor individualization focuses on open topics (JX21817902/008), and the Wu Jieping Foundation (320.6750.17006).

## Conflict of Interest

The authors declare that the research was conducted in the absence of any commercial or financial relationships that could be construed as a potential conflict of interest.
